# Modulation of fracture healing by senescence-associated secretory phenotype (SASP): a narrative review of the current literature

**DOI:** 10.1186/s40001-023-01604-7

**Published:** 2024-01-09

**Authors:** Shangkun Zhao, Zhi Qiao, Roman Pfeifer, Hans-Christoph Pape, Keya Mao, Hai Tang, Bin Meng, Songfeng Chen, Hongjian Liu

**Affiliations:** 1 Department of Orthopedic Surgery, The First Affiliated Hospital of Zhengzhou University, Zhengzhou, China; 2 Department of Traumatology, University Hospital of Zurich, Zurich, 8091 China; 3 Chinese PLA General Hospital Beijing, Beijing, 100853 China; 4 Beijing Friendship Hospital, Beijing, 100050 China; 5 First Affiliated Hospital of Soochow University, Suzhou, 215006 Jiangsu China

**Keywords:** Fracture healing, Bone, Senescence, SASP, Senolytics

## Abstract

The senescence-associated secretory phenotype (SASP) is a generic term for the secretion of cytokines, such as pro-inflammatory factors and proteases. It is a crucial feature of senescent cells. SASP factors induce tissue remodeling and immune cell recruitment. Previous studies have focused on the beneficial role of SASP during embryonic development, wound healing, tissue healing in general, immunoregulation properties, and cancer. However, some recent studies have identified several negative effects of SASP on fracture healing. Senolytics is a drug that selectively eliminates senescent cells. Senolytics can inhibit the function of senescent cells and SASP, which has been found to have positive effects on a variety of aging-related diseases. At the same time, recent data suggest that removing senescent cells may promote fracture healing. Here, we reviewed the latest research progress about SASP and illustrated the inflammatory response and the influence of SASP on fracture healing. This review aims to understand the role of SASP in fracture healing, aiming to provide an important clinical prevention and treatment strategy for fracture. Clinical trials of some senolytics agents are underway and are expected to clarify the effectiveness of their targeted therapy in the clinic in the future. Meanwhile, the adverse effects of this treatment method still need further study.

## Introduction

The phenomenon of cellular senescence in vitro was first reported in 1961 by Leonard Hayflflick [1], who found that human fibroblasts could not continue to expand after multiple passages, although they were still metabolically active. This contradicts the idea put forward by Carrel that cell tissue could survive permanently in vitro culture [2]. In recent decades, much progress has been made in the research field of cellular senescence, and many interesting biological phenomena can be attributed to cellular senescence. Many diseases were found associated with the accumulation of senescent cells, including cancer, atherosclerosis, and osteoarthritis. Senescence-associated secretory phenotype (SASP) is a generic term for bioactive molecules secreted by senescent cells that induce inflammation through autocrine and paracrine pathways and transmit senescence signals to neighboring cells, exacerbating telomere dysfunction and accelerating cellular senescence through tissue microenvironment, ROS-mediated pathways.

Studies have noticed the role of SASP during fracture healing. An analysis of public data displayed that markers of aging and SASP do increase during fracture healing and that fracture healing time is reduced after using drugs in mice to remove senescent cells [3]. One study reported that accelerated fracture healing was observed in aged mice after partial neutralization of TGF-β with TGF antibodies [4]. In several studies, the modulatory role was found to be displayed at systemic and cellular levels [5–8]. This literature aims to speculate on the effect of SASP produced by senescent cells on fracture healing.

## Senescence and SASP

### Cellular senescence and SASP

Cellular senescence is the cellular response to apoptosis, metabolic changes, and exogenous or endogenous stress. During cellular senescence, the stress or damage response can be triggered by telomere shortening, oxidative stress, oncogene activation, and DNA damage that causes cells to enter a largely irreversible cell cycle arrest and ignore apoptotic signals.

Senescent cells are still metabolically active, and most of them can secrete many cytokines, chemokines, and other bioactive molecules, collectively known as SASP or SMS [9]. Although many studies have identified SASP components in different cell types, the exact composition of SASP remains elusive and is the subject of ongoing research. However, SASP can occur differently depending on the type of senescent cells, aging triggers, and changes over time [9, 10]. SASP includes a variety of bioactive factors, which can enhance self-senescence and affect the local microenvironment of senescent cells or even the whole individual [11, 12]. SASP factors reinforce growth arrest in an autocrine manner and alter the behavior of surrounding cells in a paracrine manner [11]. Hundreds of SASP have been reported, among which the most common of factors include interleukin IL-1a, IL-6, and IL-1, and others such as IFN-γ, VEGF, ICAM-1, and GM-CSF [10, 13, 14], and some are released in the form of the exosome [14, 15].

SASP is one of the typical features of senescent cells. Identification of SASP can be used as one of the methods to assess cellular senescence. The next section reveals the methodologies in detail. Although there are some qualitative and quantitative differences in SASP in different tissues and aging models, however, based on the different modes of action of SASP activity, SASP can be classified into three types, receptor-requiring (IL-1, IL-1β, IL-6, IL-8, CXCL-1, CXCL-3, CXCL-10, HGF, TGF-β, GM-CSF, etc.); direct-response (MMPs, ROS, etc.); and regulatory (TIMPs, IGFBPs, etc) [10, 12, 14, 16–18]. There are also some SASP released as soluble molecules or exosomes. SASP has many functions, both beneficial and harmful, as shown in Table 1. Targeting senescent cells and their SASP may provide a novel strategy for the treatment of age-related diseases, such as Alzheimer’s disease (AD). These methods of using drugs to antagonize the harmful extracellular impact of senescent cells are called senomorphics [11, 19, 20]. The specifics of this study are detailed below.

**Table 1:** Functional list of senescence-associated secretory phenotype (SASP)

Marker	Function	References
AREG	Maintain immune cell function	[115]
CCL2	Recruit monocytes and macrophages	[116]
CCL5	Promote carcinogenesis	[117]
CCL27	Inhibit immune cell function	[118]
CCN1	Induce fibroblast senescence and restricts cutaneous fibrosis	[119]
FGF4	Embryonic development	[120]
FGF8	Embryonic development	[120]
GM-CSF	Regulate tumor immunosuppression	[121]
GRO	Promote tumor cells invasion	[115]
HGF	Maintain the function of stem cells	[122]
IFN-γ	Induce the activation of macrophages	[123]
IGF-1	Promote tumor cells invasion; Promote angiogenesis	[124]
IGF-2	Promote tumor cells invasion; Promote angiogenesis	[125]
IGFBP-4	Promote cellular senescence	[8]
IGFBP-6	Inhibit cellular senescence	[115, 126]
IGFBP-7	Promote cellular senescence	[127]
IL-1	Inhibit B lymphocyte formation; Involved in signaling pathways regulating SASP secretion; Lead inflammation and promote stem cell senescence	[128]
IL-6	Affect tumor cell invasion; Pluripotent stem cells; Lead to inflammation; Promote osteoclast function	[129–131]
IL-7	Associated with B lymphocyte production; Beneficial to BMSCs function	[132]
IL-8	Promote tumor cells invasion	[7]
MMP-1	Accelerated osteogenic differentiation of BMSCs	[133]
MMP-2	Embryonic development; Promote angiogenesis	[134]
MMP-3	Extracellular matrix degradation and thinning of the fibrous cap in the artery	[135]
MMP-9	Degradation of extracellular matrix; Embryonic development	[136]
MMP-13	Promote tumor cells invasion; Promote angiogenesis	[137]
PDGF-AA	Accelerated wound healing	[138]
TGF-β	Promote angiogenesis; Promote BMSCs recruitment; Regulate the RANKL/OPG ratio	[92, 139]
TNF-α	Lead to inflammation; Promote osteoclast function	[140, 141]
VEGF	Promote angiogenesis	[142]

### Validation of senescent cells

**Fig. 1:**
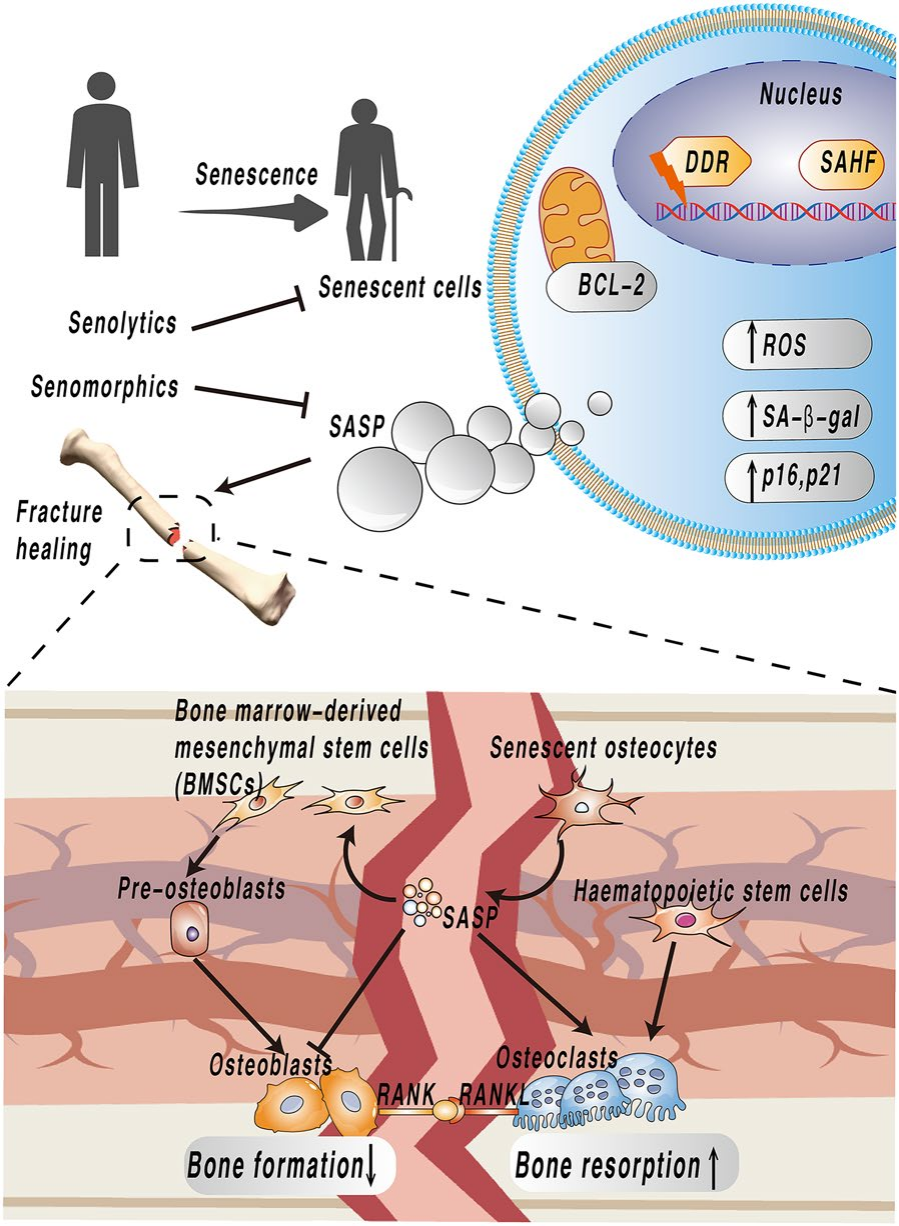
DDR occurs after DNA damage, leading to cellular senescence. Senescent cells have several features: upregulation of the BCL-2 anti-apoptotic protein family (induces resistance to apoptosis, cells undergo oxidative damage (elevated ROS can be detected), metabolic changes (including the presence of SA-β-gal aggregates, SAHF and SASP), cell cycle arrest (p21 and p16 upregulation). Osteoblasts senesce in response to stress stimuli, and these cells likely cause an inflammatory microenvironment in bone by secreting SASP effects that disrupt bone formation and enhance osteoblast function. SASP can promote aggregation of BMSCs, which can differentiate into pre-osteoblasts and osteoblasts. HSCs can differentiate into osteoclasts BMSCs, bone-marrow-derived mesenchymal stem cells. DDR, DNA damage response. RANK, receptor activator of nuclear factor Kappa-B. RANKL, RANK ligand. ROS, reactive oxygen species. SAHF, senescence-associated heterochromatin foci. *SASP* senescence-associated secretory phenotype*, SA-β-gal* senescence-associated β-galactosidase. *HSC* haematopoietic stem cell

Senescent cells have multiple phenotypes, which vary depending on their origin, but cells can be identified based on common characteristics. Due to the phenotypic heterogeneity, senescent cells cannot be identified with only one marker, but a combination of multiple senescent cell markers is needed. Some studies suggest validating at least three different traits (Fig. 1), including the (a) arrest of cell cycle progression, (b) associated structural changes, and (c) other traits known to be specific to the senescent cells being experimented with [9]. Cells can be identified as being in proliferative arrest by detecting activation of the p16–pRB axis, p53–p21 axis, or cellular proliferation and DNA replication assays. One of the characteristics of senescent cells is an increase in lysosomal content, resulting in lysosomal β-galactosidase (β-gal) activity (also known as senescence-associated β-gal or SA-β-gal. [9]. 5-Bromo-4-chloro-3-indolyl-β-D-galactopyranoside (X-gal) is the most common substrate for SA-β-Gal activity, which is catalyzed by SA-β-Gal to galactose and 5-bromo-4-chloro-3-hydroxyindole-1, which then dimerizes to form the blue precipitate indigo [21]. Senescent cells can also be identified by detecting DNA segments with chromatin alterations reinforcing senescence (DNA–SCARS), ROS, or telomere shortening. Cellular senescence can also be demonstrated by explicitly identifying the secretion of relevant SASP molecules. Interestingly, studies have found that macrophages can also express p16INK4a and SA-β-Gal in response to immune responses [22]. To identify senescent cells more accurately, multiple assays need to be used. If future studies can identify unique or standard markers of senescent cells, this will accelerate the development of cellular senescence-related research. Current fluorescent tracers and advanced imaging tools allow real-time monitoring of the aging process in vivo and real-time assessment of therapeutic effects [23–26]. A paper reports on the design of a two-parameter recognition fluorescent probe for precise imaging of cellular senescence that allows high-contrast imaging of senescence independent of cell source or type of stress [27]. These will enable the better translation of research results into clinical applications.

### The regulation of SASP

The regulation of SASP is complex and involves many factors. IL-1a is an upstream regulator of other SASP and regulates the secretion of other SASP [8, 16, 28]. NF-κB signalling regulates the senescence-associated secretory phenotype (SASP) and together with the transcription factor C/EBPβ, co-activates promoters of SASP genes. Some SASP can enhance the transcription of SASP such as IL-1a, IL-6, and IL-8 by regulating the activities of NF-κB and CEBPβ. IGFBP3, IGFBP4, and IGFBP7 are critical players in SASP and are thought to mediate cellular senescence through paracrine signaling [5–8].

#### The DNA damage response and SASP

The accumulation of DNA damage caused by replicated cells can cause DNA damage response (DDR), which can directly cause cell cycle arrest. DDR can also induce cell senescence and secrete SASP by activating two major kinase systems, ataxia telangiectasia mutant (ATM) and ATM and rad3-related (ATR), which ultimately phosphorylate p53 [29]. ATM and/or ATR are well-known upstream molecules of checkpoint kinase (CHK)1/CHK2. Stress-induced activation of MAPK and p38 was also shown to be sufficient to trigger cell senescence and SASP [30].

#### Regulation pathway of SASP translation

Nuclear factor κB (NF-κB) is an essential component in the regulatory pathway of SASP. The regulatory effects of ATM, IL-1a, and p38 on SASP are achieved by changing the activity of NF-κ B [11, 31]. NF-κB can bind to the transcription factor CCAAT/enhancer-binding protein-β (CEBPβ) and activate the promoter of the SASP gene [32], such as those that encode chemokines. The transcription factor GATA 4 can activate some genes of SASP, including IL-6, IL-8, CXCL 1(GROα), C-CSF, and ECM protease [33]. As an intermediate mediator of DDR signals and NF-κB activation, GATA 4 can regulate NF-κB activity by increasing the expression and secretion of IL-1 A and ultimately affect the secretion of SASP [34]. JAK/STAT pathway is highly activated in senescent cells and is one of the main ways to regulate cytokine production, which is related to the expression of IL-6, IL-8, IL-1A, CXCL-1, CXCL-2, CXCL-5, CXCL-6 and CXCL-10 [35]. HMGB factors constitute a family of non-histone architectural proteins that contain a distinct DNA-binding domain. HMGB2 directly binds and precisely regulates the expression of the SASP gene in oncogene-induced senescent cells [36]. The mTOR mediates cell responses to stresses, such as DNA damage and nutritional deficiencies. mTOR can activate the NF-κB pathway by promoting the translation of IL-1 A, thus affecting the secretion of SASP, including IL-6 and IL-8. On the other hand, the transcription of many SASP factors is influenced by the MAPKp38 pathway [37].

#### The innate immunity and SASP

cGAs (cGMP–AMP synthase) is a DNA sensor located in the cytoplasm of non-splinter cells, which can activate the innate immune response and lead to cell senescence. The absence of cGAs accelerated the spontaneous immortalization of mouse embryonic fibroblasts and eliminated SASP induced by natural aging and DNA damage agents [38, 39]. The activated cGAs generate a second messenger ring nucleotide (cGAMP) that binds to the stimulator of interferon genes (STING) and promotes the aggregation of Sting with TANK-binding kinase 1(TBK 1) and IκB kinases. Therefore, interferon regulatory factor 3(IRF3) and NF-κB are activated, respectively, resulting in the production of type I interferon and SASP factors (such as IL-6, IL-8, IL-1 β, and MMP-12) [7, 40, 41].

#### Other regulation methods

Recent studies have documented that extracellular nicotinamide phosphoribosyltransferase (ENAMPT) is also a component of SASP and is related to the activation of p53/p21 by NAD/NADH caused by mitochondrial damage, leading to cell senescence [42]. It has been confirmed that high-mobility group box 1protein (HMGA1) is involved in the regulation of the secretion of pro-inflammatory SASPs (IL-6, IL-8, etc.) through the regulation of NAMPT and ultimately the NAD/NADH ratio [43], and that pro-inflammatory SASP has a pro-tumorigenic effect [44]. Moreover, in mouse embryos overexpressing the HMGA1 P6 pseudogene, the mice grew faster and aged later [45]. These are associated with the activation of the p38MAPK pathway [43, 46]. The secretion of SASP can be regulated using components of the drug action regulation pathway to treat diseases, such as tumors, diabetes, and tendinopathy [11, 47].

## The inflammation of fracture healing in generally population

Bone is composed of bone extracellular matrix proteins, inorganic minerals, and a variety of resident cell types. It is an essential part of the locomotor system [48]. There are two methods of fracture healing: direct (primary) and indirect (secondary). Direct fracture healing is rare, and the fracture end needs to meet the conditions of anatomic reduction and complete the structural remodeling through direct intramembranous osteogenesis without forming calluses. Indirect fracture healing is the most common form, including endochondral and intramembranous ossification. According to the time sequence, fracture healing can be divided into hematoma formation, fibrocartilaginous callus formation, bony callus formation, and bone remodeling [49].

### Inflammation at the fracture healing site

During the early course of fracture healing, hematomas form, and in the first 48 h, an inflammatory response occurs at the fracture site, manifested by invasion of macrophages, polymorphonuclear leukocytes, and lymphocytes. After the injury, a large amount of danger-associated molecular pattern (DAMP) appears. The ruptured vasculature and exposed bone marrow cause inflammatory cell infiltration at the injured site, and fracture-related hematoma (dense cell mass) is formed by a variety of inflammatory cells (neutrophils, macrophages, T cells, B cells, regulatory B-cell mast cells.) and platelets and red blood cells [50]. The hematoma helps to initiate healing and provides the foundation for bone tissue formation. The loss of hematoma may delay the healing of the fracture. Mesenchymal stromal cells (MSCs) and hematopoietic stem cells have multidirectional differentiation potential and are closely associated with fracture healing.

Inflammatory cells, such as granulocytes and macrophages, secrete a variety of cytokines and growth factors (IL-1, IL-6, TNF-α, inducible nitric oxide synthase, transforming growth factor-β, platelet-derived growth factor, insulin-like growth factor, and fibroblast growth factor 2) [51], which are an essential part of the signaling environment for fracture healing. First, under the action of proinflammatory mediators (such as growth factors, cytokines, and chemokines), neutrophils gather at the injury site after fracture and further secrete cytokines to aggregate monocytes [52]. At the same time, activated macrophages appear, peaking at 3–7 days, releasing cytokines such as IL-1β and TNF-α to stimulate fibroblast proliferation and attract other marrow cells and lymphocytes to gather at the injury site [53]. Macrophages eventually differentiate into osteoclasts under RANKL and M-CSF and are involved in fracture repair [54]. The RANKL is necessary and sufficient for the induction of osteoclast differentiation and function, and MCSF induces osteoclast proliferation. Increased osteoclast formation accelerates cartilage resorption and promotes osseointegration. Inflammatory macrophages are essential to initiate and propagate endochondral osteogenesis [55]. Macrophages are the primary immune cells that initiate and maintain the inflammatory response and are directly involved in the osteogenesis process by secreting important cytokines associated with osteogenesis. Besides participating in allergic reactions and autoimmunity, mast cells also promote wound and fracture healing [56]. They can stimulate blood vessel permeability and angiogenesis and regulate bone metabolism [57]. The histamine and VEGF induce hyperpermeability of injured blood vessels and provide a suitable environment for tissue repair [58]. Ragipoglu et al. speculated that the effect of mast cells on bone healing might be related to their recruitment of vascular endothelial cells and coordination of metabolic activities [59].

Osteoblasts and osteoclasts have been reported to have direct cell-to-cell contact with lymphocytes, indicating the regulatory role of immune cells in the late stage of fracture healing [60]. T cells can secrete RANKL to activate osteoclasts. Interestingly, the relative number of CD4( +) T cells and CD8( +) T cells changes after fracture [60]. This could be a mechanism to enhance the bone healing process in the injured bone as CD4( +) T cells have been reported to have a pronounced osteogenic role [61, 62]. Another T-cell subset, Th17 cells, secretes IL 17F to promote osteoblast growth. On the other hand, another T-cell subset (Tregs) can inhibit the function of osteoblasts and osteoclasts by secreting IL-4, IL-10, and TGF-β [63]. γδT cells, also known as inflammatory lymphocytes, respond to acute inflammatory stress signals, promote cytokine production, recruit macrophages[64], and promote the formation of osteoblasts. It has been found that the secretion of IL17A by γδT cells can stimulate the proliferation of mesenchymal progenitor cells and the differentiation of osteoblasts [65].

B cells secrete osteoprotegerin (OPG). The protein is also expressed in osteoblasts to regulate the activity of RANKL [66]. B cells positively affected osteoclasts, whereas CD8 T cells had the opposite effect. By observing the expression ratio of OPG and RANKL, Choi et al. judged the infiltration degree of T cells and B cells during bone healing and then inferred the specific stage of the healing process [67]. Regulatory B cells (Bregs) are a kind of B cells that promote endogenous bone regeneration in the initial healing stage [68]. Bregs are a newly discovered B-cell subset that promotes endogenous bone regeneration in the initial healing phase [69]. Bregs can secrete IL-10. Studies have proved that delayed healing patients downregulated B-cell IL-10 secretion early and Bregs dysfunction may be one of the reasons for delayed fracture healing [69].

### Fracture healing is achieved through the interaction and crosstalk of stem cells, immune cells, and bone cells

Direct fracture healing refers to the direct differentiation of MSCs into functional anabolic bone cells [70]. In indirect fracture healing, MSCs are transformed into chondroblasts regulated by transcription factor SRY-related high mobility group-box gene 9 (Sox9) and M2-type macrophages [71]. These cells will further differentiate into chondrocytes and finally transform into hypertrophic chondrocytes during the formation of hard callus [72]. These cells differentiate into osteoblasts under the induction of transcription factors, such as Runx2 and Sp7, etc. [73]. Bone-marrow mesenchymal stromal cells differentiate into osteoblast phenotype in the periosteum and surrounding soft tissues, and the Wnt/β-catenin pathway is involved in this process [74]. The osteoblast transcription factor RUNX2, the enzyme alkaline phosphatase, and other mineralization proteins can mineralize bone matrix [75]. Osteoclasts are large, multinucleate cells that secret acid and proteolytic enzymes to dissolve the bone matrix. Nuclear factor Kappa-B (RANK) ligand (RANK-L) is an anchored cell membrane factor that can interact with RANK as its receptor and eventually induce osteoclast precursor maturation [76]. Osteoblasts themselves secrete GM-CSF, which is involved in promoting osteoclast differentiation and maturation [77]. Future research will focus on understanding how multiple cell types and the resulting signaling networks integrate spatially over time to regulate healing. The inflammatory response during fracture healing is of remarkable complexity, but in silico models help us to understand the principles that regulate the various events that occur at the tissue, cellular, and subcellular levels. Silico models of the inflammatory response in bone fracture healing have been constructed to further explore the crosstalk of multiple cells in fracture healing [78].

## SASP in fracture healing

Existing studies confirmed that SASP contributes to the recovery of injured tissues [79, 80]. What is the role of SASP in skeletal injury and repair? One study analyzed SASP-related components in fracture healing tissues using qRT-PCR and found that most SASP increased rapidly during fracture healing, especially CCL7 increased 70-fold at day 14 of the fracture and Plasminogen activator inhibitor-1 (Pai1 or Serpine1) increased more than 60-fold at day 8 but decreased substantially at day 14. At the same time, interleukins showed a significant increase at the beginning of fracture healing, such as IL-6 and IL-17 [3]. The variety of SASPs the high heterogeneity of gene expression of SASPs, and the timing of their peak concentrations may be related to their effects.

### IL-6

Bone-marrow-derived mesenchymal stromal cells (BMSCs) play an essential role in fracture healing, differentiating into osteoblasts and BMSCs recruit to bone resorption sites for bone tissue remodeling. Animal studies found that SASP components such as IL-6 were secreted by SA-β-gal-positive, cell cycle-arrested senescent cells in irradiated mice and found that SASP further affected the differentiation impairment of BMSCs through paracrine signaling [81]. It has been suggested that IL-6 deficiency enhances the expression of osteoblast-related genes (Runx2 and Col1a1) and decreases the expression of osteoclast-related genes [82]. A study indicated that SASP targeting might be an effective treatment for irradiation-induced bone loss [81]. One study found more IL-6 from BMSCs from older adults than younger adults, and BMSCs can regulate osteoblast and osteoclast activity through the secretion of SASP [83]. IL-6 acts as an osteoblast inhibitor and promotes osteoclast formation [84], affecting bone remodeling and possibly fracture healing.

### TNF-α

It was reported that TNF-α, such as IL-6, has a stimulating effect on bone resorption but an inhibitory effect on bone formation [84, 85], which may be related to the regulation of RANKL [86]. Some studies confirmed that RANKL-dependent pathways activated by pro-inflammatory cytokines can induce osteoclast formation, therefore, enhancing osteoclast activity [87, 88]. TNF-α can also indirectly increase RANKL expression and affect osteoclast differentiation by activating osteoblasts, B cells, and T cells [88]. One study in mice has found that p12 mediates the bone inhibition of TNF-α and TNF-β, and it is found that the use of TNF-α and TNF-β blockers in the aged mouse model can accelerate the healing of mouse fractures [89]. It has also been suggested that aging leads to increased long-term expression of TNF-α, which leads to delayed early inflammation and cartilage formation processes in fracture healing and a decrease in overall VEGF, which affects angiogenesis to the point of affecting bone healing, as well as being one of the main reasons why diabetes affects bone healing in mice [90]. In contrast, Glass et al. found that low concentrations of TNF-α could promote bone healing by enhancing the recruitment and differentiation of muscle-derived stromal cells [91].

### TGF-β

TGF-β is also an integral component of SASP, and elevated markers of cellular senescence and enhanced TGF expression were observed at fracture sites in mice. TGF-β1 can potentially promote the recruitment of MSCs [92], which are involved in fracture healing upon differentiation. Scholars have reported that TGF-β contributes to treating bone defects in rats [93]. Some scholars have observed that bone samples from aged mice and humans have high levels of TGF-β and can stimulate the breakdown of TNF receptor-associated factor 3 to inhibit the differentiation of mesenchymal progenitor cells into osteoblasts [94]. High levels of TGF-β were detected in the blood of both mice and humans with fracture nonunion [95]. One study reported that accelerated fracture healing was observed in aged mice after partial neutralization of TGF-β with TGF antibodies [4]. However, TGF-β has also been identified to contribute to the angiogenesis of fracture-healing tissue. During osteoclastogenesis, RANKL drives osteoclast differentiation, while OPG antagonizes RANKL action, and the RANKL/OPG ratio affects the osteoclast differentiation rate and bone resorption process [96, 97]. Different concentrations of TGF-β have different effects on the RANKL/OPG ratio and ultimately on osteoclast differentiation (low concentrations of TGF-β increase the stimulation of osteoclast differentiation, while high concentrations of TGF-β have the opposite effect) [98, 99].

#### Other SASP factors

The differentiation process of BMSCs is mediated by IL-8, and MMP [100, 101], which are also the SASP molecules, which may affect the differentiation outcome of BMSCs, which may be one way in which SASP affects fracture healing and bone loss. The function of SASP in skeletal tissues needs further experiments. IL-8 and MMP9 may affect the activity of these cells, which are critical participating cells in the bone healing process, in the manner described above and may, therefore, influence the bone healing process (Fig. 1). There are relatively few experimental studies addressing SASP on fracture healing, and a recent study performed computer analysis of public mRNAseq data found that senescence and SASP were associated with fracture healing [3]. The currently available results demonstrate that the effects of SASP on the skeletal system are primarily detrimental. However, transient SASP was found to promote tissue recovery in wound healing and liver fibrosis. In contrast, chronic SASP had unfavorable effects on tissues [102, 103], and it has been speculated that there may be a threshold beyond which SASP would have different effects [3]. The local effects of senescence, including those of SASP, may be related to the abundance and duration of senescent cell burden. The accumulation of senescent cells leads to adverse effects, negatively correlated with local immune clearance.

However, the onset of cellular senescence in healing bone is also a short-time course, but the bone healing-promoting effect of SASP was not observed in the available experiments. This may be because the highly inflammatory state [104] after fracture masks the positive impact of transiently appearing senescent cells. On the other hand, bone, unlike other tissues, such as skin viscera, is the only tissue in the organism that is fully recoverable and does not form scars. We can then wonder whether the mechanism of SASP repair of injured tissues interacts with the mechanism of scar formation. This question needs to be addressed by further research explorations.

## Senolytics and fracture healing

Numerous findings have shown that senescent cell removal is largely beneficial, leading to an exponential advance in research on therapeutic strategies for senescence depletion (called senotherapy) [105]. A variety of drugs with the ability to eliminate senescent cells have now been reported. These drugs can selectively target senescent cells (called senolytics) or selectively inhibit SASP (called senomorphics) from reducing or suppressing senescent cells in the organism [11] (Fig. 1). Some researchers have classified senolytics into three major classes: Class I senolytics are BCL-2 family protein inhibitors whose inhibition culminates in the selective apoptosis of senescent cells [106], such as ABT-737 and ABT-263 (also known as navitoclax) [11]; Class II senolytics can inhibit pro-survival signals upstream of senescent cells to resist cell death. Such as the senolytic peptide FOXO4–DRI acts by interfering with the binding of FOXO4 and p53 [107], as dasatinib and quercetin act by downregulating AKT signaling [108], HSP90 inhibitors also act as senolytics by mediating the downregulation of AKT signaling [109], and the mTOR inhibitor rapamycin acts by affecting NF-κB [110]; Class III senolytics can interfere with the intracellular homeostasis of senescent cells, such as piperlongumine, and procyanidin C(add source). Senomorphics can inhibit the extracellular function of senescent cells by targeting senescence-associated signaling pathways (e.g., MAPK, NF-κB, mTOR) while maintaining the survival of senescent cell (add source)s.

Some projects that used senolytics (dasatinib and quercetin) in aged mice with established bone loss have observed a reduction in senescent osteoclasts [111], contributing to the treatment of osteoporosis. However, some studies reported trabecular bone loss and impaired bone formation in BMSC in aged mice after using Navitoclax (ABT-263) [112]. A study reported that intermittent treatment of young fracture mice with dasatinib and quercetin resulted in a downregulation of aging markers in fracture healing tissue and contributed to fracture healing [3]. In contrast, short-term treatment of fracture mice with dasatinib and quercetin (1, 3, 5, and 7 days after fracture) revealed that accelerated fracture healing was observed only in the older mice [4]. Several clinical trials are currently underway: dasatinib and quercetin are testing for bone health (NCT04313634), fisetin is testing for skeletal health (NCT04313634), and osteoarthritis (NCT04210986).

The relationship between senolytics and fracture healing continues to be studied, and there are no precise rules for the dose and duration of treatment. Senolytics can also cause some adverse effects. Some studies have found that some senolytics may cause severe reductions in platelets or neutrophils [112, 113]. The drug causes massive apoptosis of senescent cells, which may lead to tissue atrophy [114]. Although the mechanism by which the adverse effects of senolytics occur is not yet clear, existing studies suggest that long-term use of senolytics drugs may lead to additional side effects. The safety of senolytics will be the focus of the next studies. The safety of senolytics will be the focus of further research.

## Conclusions

Senescence is a state of cellular proliferative arrest as senescent cells secrete SASP and exert paracrine effects, affecting neighboring cells. The effects of senescence on fracture healing involve the four stages of fracture healing. Fracture healing is a complex process involving multiple cells and molecules, and the inflammatory response affects the quality of fracture healing. SASP can affect inflammatory molecules in fracture healing and multiple cellular components involved in healing, but the mechanisms are not yet precise. In addition, senolytics are potentially effective in treating non-healing fractures, but the specific dosing method still needs to be simpler. In conclusion, further studies are needed to investigate the effects of SASP on fracture healing and to assess whether treatment targeting SASP will promote fracture healing.

## Data Availability

The authors can be contacted for data requests.

## Abbreviations

ATM: Ataxia telangiectasia mutant
ATR: ATM and rad3-related
CEBP: CCAAT/enhancer-binding protein
CHK: Checkpoint kinase
CXCL: C–X–C motif chemokine ligand
DDR: DNA damage response
ECM: Extracellular matrix
EVs: Extracellular vesicles
G-CSF: Granulocyte colony-stimulating factor
GM-CSF: Granulocyte–macrophage colony-stimulating factor
HMGB: High mobility group box
ICAM: Intercellular adhesion molecule
IFN: Interferon
IGFBP: Insulin-like growth factor binding protein
IL: Interleukin
JAK/STAT: Janus kinase/signal transducer and activator of the tran-ions
MAPK: Mitogen-activated protein kinase
MMP: Matrix metalloproteinase
mTOR: Mechanistic target of rapamycin
NF-κB: Nuclear factor κB
ROS: Reactive oxygen species
SASP: Senescence-associated secretory phenotype
SMS: Senescence-messaging secretome
TNF: Tumor necrosis factor
VEGF: Vascular endothelial growth factor
